# Roles of Macrophage Migration Inhibitory Factor in Dengue Pathogenesis: From Pathogenic Factor to Therapeutic Target

**DOI:** 10.3390/microorganisms8060891

**Published:** 2020-06-12

**Authors:** Yen-Chung Lai, Chiao-Hsuan Chao, Trai-Ming Yeh

**Affiliations:** 1 Institute of Basic Medical Sciences, College of Medicine, National Cheng Kung University, Tainan 70101, Taiwan; hityouok@livemail.tw (Y.-C.L.); sheex5050@hotmail.com (C.-H.C.); 2 Department of Medical Laboratory Science and Biotechnology, College of Medicine, National Cheng Kung University, Tainan 70101, Taiwan

**Keywords:** Dengue virus, dengue pathogenesis, macrophage migration inhibitory factor, autophagy

## Abstract

Dengue virus (DENV) infection is the most prevalent mosquito-borne viral infection and can lead to severe dengue hemorrhagic fever (DHF) and even life-threatening dengue shock syndrome (DSS). Although the cytokine storm has been revealed as a critical factor in dengue disease, the limited understanding of dengue immunopathogenesis hinders the development of effective treatments. Macrophage migration inhibitory factor (MIF) is a pleiotropic proinflammatory cytokine that mediates diverse immune responses, and the serum level of MIF positively correlates with disease severity in patients with dengue. MIF is involved in DENV replication and many pathological changes, such as vascular leakage, during DENV infection. In this paper, the pathogenic roles of MIF and the regulation of MIF secretion during DENV infection are reviewed. Furthermore, whether MIF is a potential therapeutic target against DENV infection is also discussed.

## 1. Introduction

Dengue virus (DENV) infection is the most widespread mosquito-borne viral infection in the tropics and subtropics. It is estimated that DENV causes 390 million infections yearly, even though more than 50% of cases of DENV infection are asymptomatic or cause mild flu-like illness, including fatigue, headache, myalgia and nausea with sudden onset of fever called dengue fever (DF). Unfortunately, one in twenty infected individuals may suffer from a more severe illness, which is termed dengue hemorrhagic fever (DHF), and even develop life-threatening dengue shock syndrome (DSS) [1]. In 2009, the WHO issued a new guideline that classifies symptomatic cases as nonsevere dengue or severe dengue according to disease severity. Nonsevere dengue cases can be further divided into two subgroups: patients with warning signs and those without warning signs. The warning signs include abdominal pain or tenderness, persistent vomiting, clinical fluid accumulation, mucosal bleeding, lethargy, restlessness, liver enlargement and decreased platelet counts. The criteria for severe dengue include severe plasma leakage, severe bleeding, and severe organ involvement [2]. The mortality of DF is less than 1%; however, severe dengue may carry a mortality rate up to 26% [3]. Although there is one vaccine (Dengvaxia^®^) that has been licensed to prevent dengue in several countries, this vaccine rendered only partial protection against DENV2 infection. In addition, the vaccine is associated with an unexplained increased incidence of hospitalization for severe dengue disease in seronegative vaccine recipients [4,5]. Furthermore, due to the limited understanding of the exact pathogenic mechanisms to cause vascular leakage, no effective therapeutic drugs are available, and the treatment for hospitalized dengue patients is still supportive care and fluid replacement [6]. As a result, it is important to further understand the pathogenesis of dengue to develop an effective treatment against DENV infection.

DENV is an enveloped positive-stranded RNA virus that contains three structural proteins in the mature virion: envelope (E), membrane (M) and capsid (C). Based on antigenic differences in the E protein, DENV can be divided into four different serotypes (DENV1, DENV2, DENV3, and DENV4). During the initial step of DENV infection, an infectious virion enters the cell via attachment to a distinct cell surface receptor, such as DC-SIGN, GRP78, mannose receptor, heparan sulfate, TIM receptors, and Claudin-1 [7]. Upon internalization by receptor-mediated endocytosis, the low pH environment triggers a conformational change in the E dimer and fusion with the endosomal membrane, which is followed by uncoating of the nucleocapsid and release of the viral genome into the cytoplasm. The viral RNA is translated into a single polyprotein that is associated with the endoplasmic reticulum (ER) and processed into three structural proteins, as well as seven nonstructural (NS) proteins (NS1, NS2A, NS2B, NS3, NS4A, NS4B, and NS5), by viral and cellular proteases. Upon polyprotein translation and folding of each viral protein, the NS proteins facilitate replication of the viral genome. Newly synthesized RNA is packaged into a nucleocapsid and covered by a precursor membrane (prM)/E heterodimer. The virion assembles in the ER and transits to the Golgi. During the viral maturation process, prM is cleaved into M by the cellular endoprotease furin in the acidic secretory system. After dissociation of the Pr peptide, mature virions that are able to infect new cells are exocytosed [8].

The antibody-dependent enhancement (ADE) theory of dengue pathogenesis was first proposed by Halstead in 1977 [9] to explain the phenomenon in which secondary infection with different serotypes of DENV may cause more severe disease development. According to the ADE theory, antibodies (Abs) stimulated by the primary infection can only partially bind to different serotypes of DENV (heterotypic DENV virion) and cannot fully neutralize infection. Instead, these subneutralizing Abs facilitate viral entry by binding with Fc receptors on immune cells, which results in robust infection. However, not all severe dengue cases are secondary DENV infections. On the other hand, virus virulence theory has been proposed to explain the occurrence of DHF/DSS might correlate with the introduction of more virulent DENV strains in distinct areas [10,11,12]. Yet, age has also been indicated to influence the disease severity independent of virus virulence in some studies [13,14]. Therefore, the interplay of viral and host factors may be crucial determinants of severe dengue development.

The association of cytokines in dengue patients with severe dengue has been widely studied [15]. Furthermore, cytokine storms have been found in patients with severe dengue who show high levels of cytokines and chemokines in circulation, accompanied by vascular leakage, hemorrhagic symptoms, and shock [16,17]. During the early febrile phase, the first wave of antiviral cytokines, such as type I interferon (IFN), and proinflammatory cytokines, such as macrophage migration inhibitory factor (MIF), monocyte chemotactic protein 1 (MCP-1), interleukin (IL)-6, and IL-8, are produced by initially exposed cells, such as epidermal dendritic cells and endothelial cells, and these factors can be released immediately [15,18,19,20,21]. Subsequently, cytokine-induced innate immune cells, such as natural killer (NK) cells and invariant NKT cells, can secrete IL-15 or IFN-γ and IL-4, which recruit more adaptive immune cells and induce cytokine release [22,23]. At the time of plasma leakage, elevated levels of permeability-enhancing factors, tumor necrosis factor-α (TNF-α) and vascular endothelial growth factor-A (VEGFA) are found in severe dengue patients and contribute to three main pathological features: plasma leakage, hemorrhage and coagulopathy [24,25]. However, it is unclear which proinflammatory cytokines dominate the cytokine storm upon DENV infection. Among these cytokines, MIF is the only cytokine that is preformed inside cells and can be secreted very early during infection. Additionally, MIF can promote the synthesis of proinflammatory cytokines, such as TNF, IFN-γ, IL-1β, IL-2, IL-6, and IL-8 [26], to further amplify the production of other proinflammatory cytokines. Therefore, in this review, we will focus on the pathogenic roles of MIF during DENV infection and discuss whether it can be a therapeutic target against DENV infection.

## 2. MIF Expression and Function

MIF, a 12.5 kDa protein was first identified as a cytokine that is mainly released from T cells upon antigen stimulation and inhibits the random migration of macrophages [27]. Later, MIF was found to be widely distributed in various immune and nonimmune cells, including macrophages, platelets, endothelial cells, epithelial cells that mediate several immune responses with pluripotent activity. Unlike most cytokines, MIF is constitutively expressed and stored in preformed “intracellular pools” during normal homeostasis [28], which means it can be released from cells upon inflammatory and stress stimulation promptly. Accordingly, MIF secretion can be found even without de novo synthesis. Since MIF does not possess an *N*-terminal secretory sequence, MIF is released from cells via nonconventional ER/Golgi secretory pathways [29]. Both intracellular and extracellular MIF exhibit pluripotent functions. Intracellular MIF can modulate AP-1 activity and the cell cycle by interacting with cytosolic Jun activation domain binding protein 1 (JAB-1) [30,31]. Overexpression of endogenous MIF significantly suppresses p53-dependent and oxidative stress-induced apoptosis [32,33]. On the other hand, secreted MIF can bind to some distinct cell receptors, such as CD74 and its coreceptors CD44, CXCR2, CXCR4 and CXCR7, which activate several signaling pathways, such as the ERK1/2 and PI3K/AKT pathways, which are responsible for cell proliferation, survival, and immune regulation [34,35,36]. In addition, MIF also has endocrine activity to regulate insulin secretion [37] and tautomerase activity to manipulate cell growth [38].

Under normal physiological conditions, MIF is constitutively expressed at a low level between 2–10 ng/mL, while the MIF concentration in human plasma fluctuates in response to stress stimuli such as sepsis, infection and different inflammatory disorders [39,40,41]. High concentrations of MIF can activate the synthesis of more proinflammatory cytokines, such as TNF, IFN-γ, IL-1β, IL-2, IL-6, and IL-8 [26], which accelerates inflammatory disease development. Furthermore, MIF also contributes to the pathogenesis of various viral infections, including human immunodeficiency virus (HIV), respiratory syncytial virus (RSV), West Nile virus (WNV) and DENV [42,43,44,45,46].

## 3. Pathogenic Roles of MIF in Dengue Pathogenesis

**Figure 1:**
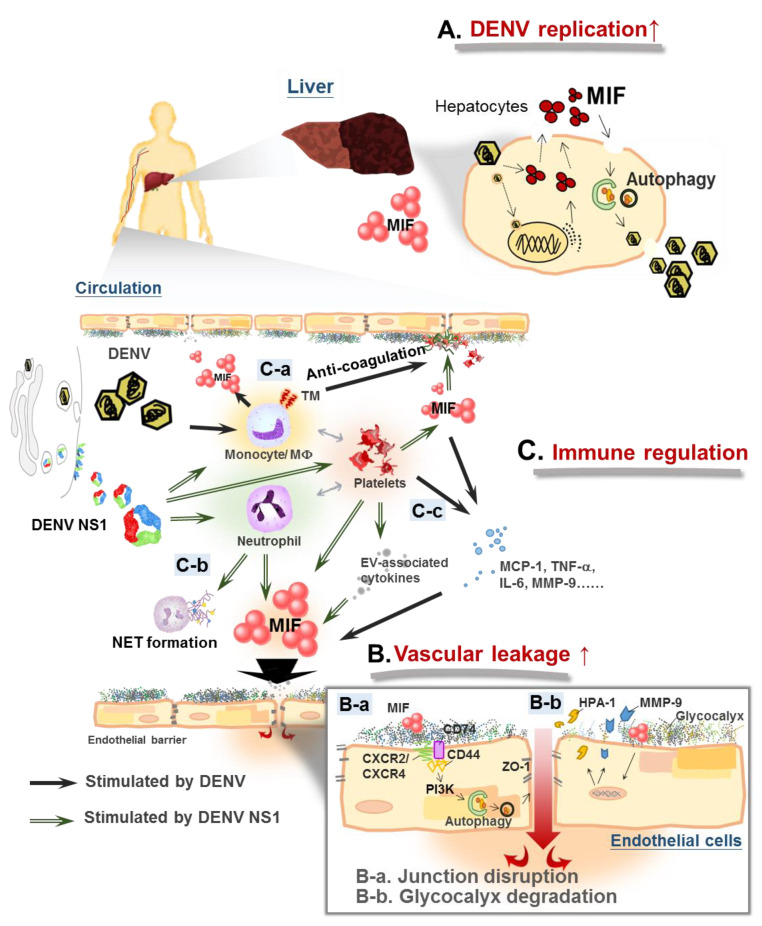
Macrophage migration inhibitory factor (MIF) mediates immune response crosstalk in dengue pathogenesis. (**A**) Dengue virus (DENV) infection induces MIF expression and secretion in epithelial cells. MIF facilitates dengue virus (DENV) replication through stimulating autophagy. (**B**): a. DENV infection enhances vascular permeability by disrupting the distribution of the endothelial tight junction protein zonula occludens-1 (ZO-1) through MIF-activated phosphatidylinositol-3-kinase/mitogen-activated protein kinase kinase-extracellular signal–regulated kinase/c-Jun *N*-terminal kinase (PI3K/MEK-ERK/JNK) signaling pathways; DENV nonstructural protein 1 (NS1) causes disarray of the endothelial tight junction through MIF-induced autophagy, b. DENV NS1-induced MIF secretion triggers the release of heparanase 1 (HPA-1) and metalloproteinase 9 (MMP-9), enhancing glycocalyx shedding from endothelial cells. (**C**): a. DENV infection stimulates thrombomodulin (TM) expression in monocytes through upregulation of the MIF signaling pathway, which disrupts the function of coagulation factors, b. DENV/DENV NS1 stimulates neutrophil extracellular traps (NET) formation in activated neutrophils, c. MIF regulates the interaction of leukocytes and activated platelets in DENV infection, which promotes the release of more inflammatory cytokines and permeability-enhancing factors.

The first evidence of the pathogenic role of MIF in dengue disease was indicated by the positive correlation between the MIF level in the sera of dengue patients and disease severity [47]. Later, another group also demonstrated that DENV2-induced inflammation, thrombocytopenia, viral load and disease severity could be attenuated in *Mif*^−/−^ mice [48]. In 2015, Ferreira et al. also showed that the serum level of MIF is higher in patients with DHF than DF [49]. However, the pathogenic mechanisms and source of circulating MIF were not further investigated. Here, we propose three main pathogenic roles of MIF in the immunoregulatory crosstalk during DENV infection: (1) Facilitation of DENV replication; (2) enhancement of vascular leakage; (3) regulation of the immune response (Figure 1).

### 3.1. MIF Enhances DENV Replication in Host Cells

It has been shown that DENV2 infection triggers MIF expression and secretion, which enhances DENV2 replication in HuH-7 cells [50]. Autophagy is pivotal for DENV replication [51], and MIF is required for the induction of autophagy in DENV2-infected HuH-7 cells. Blocking MIF with inhibitors or knockdown of MIF expression by shRNA inhibited both DENV-induced LC3 conversion and DENV replication. To clarify the correlation between these two parallel effects, we found that incubation with recombinant MIF (rMIF) only enhanced viral replication in MIF knockdown but not LC3 knockdown cells. These results suggest that autophagy is required for MIF-induced DENV replication in HuH-7 cells.

Given that DENV infection induces autophagy, which is required for DENV replication, the mechanisms of DENV-induced autophagy have been widely studied. The formation of autophagosomes provides a dock for the DENV replication complex, which supports viral replication in nonphagocytic cells [51]. DENV infection also induces selective autophagy associated with lipid metabolism. DENV-induced autophagosomes facilitate mitochondrial β-oxidation associated with host lipid metabolism, which enhances ATP production for viral replication [52]. In addition, NS4A expression induces autophagosome formation and inhibits cell apoptosis during DENV2 infection, which contributes to prolonged viral replication [53].

Previously, MIF was found to induce autophagy under starvation conditions. Starvation-triggered MIF binds to its receptor CD74 in autocrine or paracrine fashions, and then autophagy is induced through reactive oxygen species (ROS) generation [54]. CD74-mediated MIF endocytosis can also activate ERK phosphorylation [55], leading to autophagy [56]. In another study, it was demonstrated that live DENV2-induced endoplasmic reticulum (ER) stress is required for autophagy activation, viral replication and pathogenesis in HuH-7 and A549 cells [57]. Although the direct correlation between DENV-induced ER stress and MIF secretion has not been elucidated, both MIF and ER stress can induce autophagy through ERK1/2 phosphorylation [58,59] and ROS generation [60]. Interestingly, MIF is able to induce ER stress in a liver injury model [61]. Therefore, it is possible that MIF signal transduction may trigger ER stress and ERK activation upon DENV infection, leading to autophagy induction and viral replication, or vice versa. Although further investigation is required to understand the relationship between MIF and DENV-induced ER stress in the future, our previous results showed that DENV-induced MIF secretion can induce autophagy-facilitated viral replication, which may explain why reduced viremia was found in *Mif*^−/−^ mice than in control mice [48].

### 3.2. MIF Contributes to Vascular Leakage

In addition to the pro-viral effect in hepatocytes and the lung epithelial cells, pathogenic roles of MIF in endothelial cells were also found. MIF is crucial in the vasculature, which has been addressed in several studies. MIF expression in human endothelial cells is upregulated upon treatment with agonists that can trigger endothelial hyperpermeability, such as thrombin [62] or oxLDL [63]. On the other hand, inhibiting MIF with either inhibitors or anti-MIF antibodies could rescue thrombin-induced vascular leakage [64], suggesting that MIF plays a role in vascular leakage. Moreover, treatment with rMIF could directly increase permeability within 30 min in the dermal microvascular endothelial cell line HMEC-1 and by subcutaneous injection in a mouse model [59]. Not surprisingly, MIF has also been shown to be involved in vascular hyperpermeability upon DENV infection. DENV infection can stimulate MIF secretion from HuH-7 cells. Conditioned medium collected from DENV-infected HuH-7 cells can enhance the permeability of human endothelial cell lines by disrupting the distribution of the endothelial tight junction protein zonula occludens-1 (ZO-1) through MIF-activated phosphatidylinositol-3-kinase/mitogen-activated protein kinase kinase-extracellular signal–regulated kinase/c-Jun *N*-terminal kinase (PI3K/MEK-ERK/JNK) signaling pathways [19]. Moreover, MIF also plays an essential role in DENV NS1-triggered endothelial permeability. DENV NS1 is a viral protein that is the main reason for vascular leakage during dengue infection [65]. There are at least two mechanisms involved in DENV NS1-triggered endothelial permeability, both of which involve MIF: enzyme-dependent glycocalyx degradation and autophagy-facilitated disruption of endothelial integrity. DENV NS1 can activate MIF secretion in endothelial cells, which triggers the release of heparan sulfate-specific heparanase 1 (HPA-1) and endothelial glycocalyx shedding [66]. On the other hand, DENV NS1 can cause the disarray of endothelial tight junctions through MIF-induced autophagy [67]. Taken together, these findings suggest that MIF plays important roles in vascular leakage during DENV infection.

### 3.3. MIF Modulates the Functions of Immune Cells during DENV Infection

In addition to viral replication and endothelial hyperpermeability, MIF may also regulate the immune response of different immune cells during DENV infection. In phagocytic cells, MIF can upregulate coagulation molecules, such as thrombomodulin (TM), in DENV2-infected THP-1 cells [68]. TM can compete with fibrinogen to bind to thrombin and inhibit fibrin formation, thereby contributing to coagulopathy in dengue disease. As a pivotal mediator of inflammatory cytokines, MIF inhibition can attenuate DENV2 infection-induced TNF-α and IL-6 production, and DENV NS1-triggered metalloproteinase 9 (MMP-9) production as demonstrated in THP-1 cells [48]. As TNF-α and IL-6 are critical proinflammatory cytokines involved in vascular permeability [69,70] and MMP-9 is a key enzyme that can trim the glycocalyx layer in endothelial cells [71], upregulation of MIF in leukocytes can further contribute to DENV-induced vascular permeability. Thus, MIF can lead to coagulopathy and vascular leakage through DENV-stimulated immune cells, which may explain why the increased hematocrit was attenuated in DENV2-infected *Mif*^−/−^ mice [48]. On the other hand, neutrophil extracellular traps (NETs) induced by activated neutrophils are thought to be a pathogenic feature that amplifies inflammatory responses in dengue disease [72]. MIF is required for NET formation in immune disorders [73,74]. Therefore, the release of MIF from neutrophils may induce NET formation and inflammation in DENV infection, which contributes to dengue pathogenesis. In addition to these immune cells, activated platelets are also a source of MIF. Platelet-derived MIF is a unique chemokine that leads to monocyte adhesion on endothelial layers [75]. DENV NS1 can contribute to platelet activation and aggregation, thus enhancing platelet adhesion to endothelial cells [76]. In addition, extracellular vesicles (EVs) have been identified as functional conveyors of MIF in obesity [77], as well as crucial mediators in platelet–leukocyte interactions upon DENV2 infection [78]. Platelet-derived MIF may be involved in DENV NS1-induced thrombocytopenia and hemorrhage.

## 4. Mechanisms of DENV Infection-Induced MIF Secretion

**Figure 2:**
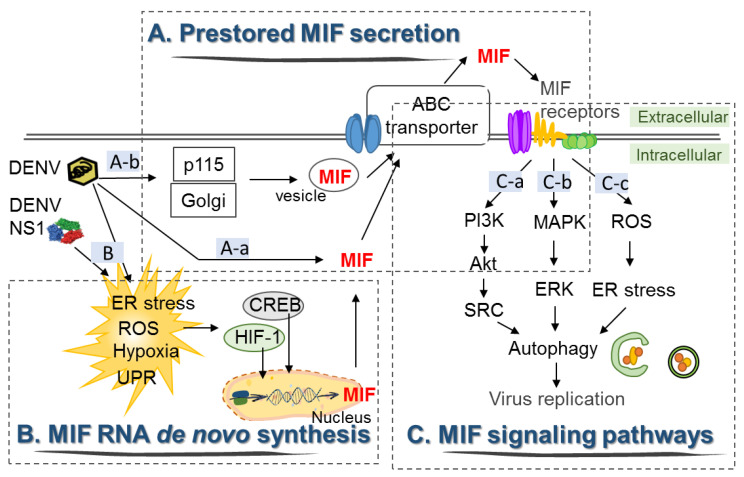
The signal transduction of DENV-induced MIF expression and secretion in HuH-7 cells. (**A**): a. DENV induces the release of MIF from intracellular pools through the ATP binding cassette (ABC) transporter, b. Golgi-associated protein p115 facilitates MIF secretion via Golgi vesicle trafficking upon DENV infection. (**B**) DENV infection induces expression of the transcription factors hypoxia-inducible factor 1 (HIF-1) and cAMP-response element-binding protein (CREB), which may drive transcription of MIF RNA. (**C**): a–c. The release of MIF initiates different signal transduction pathways by binding to its distinct membrane-bound receptors and coreceptors, which facilitates autophagy and viral replication.

Although a previous study demonstrated that serum levels of MIF are significantly higher in all DHF patients who died than in surviving DHF and DF patients [47], the main source of secreted MIF and its release mechanisms are still unclear. Here, the possible mechanisms of DENV-induced MIF secretion and expression are discussed (Figure 2).

### 4.1. DENV Induces the Secretion of Preformed MIF from Intracellular Pools

As it lacks an *N*-terminal secretory sequence, MIF is known to be released from the intracellular pools very quickly upon stimulation. It has been shown that productive DENV2 and DENV3 infection can stimulate MIF release from human macrophages and Hep G2 cells without the requirement of MIF RNA transcription at 14 h post-infection [48]. Similarly, DENV2 infection of HuH-7 cells can drive two waves of MIF secretion [50]. DENV2 infection induced the first wave of MIF secretion at 3–6 h post-infection. Since no obvious lactate dehydrogenase (LDH) release nor a significant increase in MIF mRNA occurred during this period of time, these results suggest that the first wave of MIF secretion was caused by the release of MIF from intracellular pools, and this secretion is independent of MIF gene transcription and cell death [50].

### 4.2. DENV Induces De Novo Synthesis of MIF RNA in Host Cells

However, MIF mRNA expression is increased in HuH-7 cells at 12 h to 48 h post-DENV2 infection, as shown by RT-PCR [50]. Although quantitative RT-PCR or real-time RT-PCR should be used to detect changes in the MIF RNA level more precisely, these results suggest that DENV infection can trigger signals to enhance MIF transcription. As viral RNA and viral proteins can trigger cellular oxidative stress, which is a strong stimulus for MIF secretion and expression [79], various cellular oxidative stresses, such as ER stress, the unfolded protein response (UPR), hypoxic response and mitochondrial ROS production, might be the upstream stimulators for MIF production during DENV infection [80,81]. Among them, two hypoxia-related transcription factors, hypoxia-inducible factor 1 (HIF-1) and cAMP-response element-binding protein (CREB), were found to facilitate DENV2 infection [82,83]. In addition, MIF transcription can be driven by HIF-1 in response to hypoxia or by CREB in response to ER stress. Therefore, live DENV induces a hypoxic response and ER stress, followed by MIF RNA transcription through HIF-1 or CREB activation, which may lead to the second wave of MIF secretion. However, in another study, MIF transcription was only marginally affected by DENV3 infection in the human hepatoma cell line Hep G2 [48]. A possible explanation for this discrepancy is that different infection time points and virus strains were used in these two studies. We showed that MIF transcription was gradually increased at 12 h post-infection and peaked at 24 h post-infection in HuH-7 cells, while in another study, the MIF RNA level was only analyzed within 14 h post-infection. In addition, viral infection-induced MIF secretion and production seem to be virus- and cell-dependent. For instance, influenza A virus infection does not induce MIF gene transcription but causes the release of preformed MIF from lung epithelial cells due to necrotic cell death [84]. However, infection of macrophages with Sindbis virus resulted in MIF release from intracellular pools without a significant increase in MIF transcription or cell death [48]. Accordingly, the mechanisms of MIF production and the kinetics of MIF secretion induced by DENV infection in different types of cells require further investigation.

### 4.3. Signaling Pathways by Which DENV Induces Secretion of MIF and Autophagy in Host Cells

Compared with the classical pathway of cytokine secretion, MIF can be released from preformed pools shortly after stimulation. Secretion of preformed MIF has been shown to be mediated by the ATP binding cassette (ABC) transporter [29], as well as vesicle or exosome secretory pathways [85]. In addition, DENV infection may stimulate MIF secretion mediated by the Golgi-associated protein p115 via Golgi vesicle trafficking, which is similar to the effect of bacterial infection [86]. In our study, since UV-inactivated viral particles could not induce MIF secretion or expression, it is possible that DENV infection triggered RNA sensing or pattern recognition receptor (PRR) activation, which was followed by the release of preformed MIF from the cytosol through the vesicle trafficking secretory pathway. The release of preformed MIF may further initiate many cellular signal transduction pathways through binding to membrane-expressed MIF receptors, including CD74/CD44, CXCR2, CXCR4 and CXCR7 [87,88]. Initiation of autophagy signal transduction induced by MIF binding to its receptors leads to activation of the PI3K/Akt/SRC and MAPK/ERK pathways [89,90], which may explain why the first wave of MIF secretion occurs before DENV2-induced autophagic flux in HuH-7 cells [50].

## 5. MIF as a Therapeutic Target against DENV Infection

To reduce the burden of dengue disease, effective drugs to treat DENV infection are urgently needed until a satisfactory vaccine becomes available. Based on the proposed dengue pathogenesis described above, current DENV therapeutic development has focused on two major targets: (1) viral factors and (2) host factors.

By targeting viral factors, many approaches to protect against different viral life cycles have been tested, such as inhibition of receptor-dependent viral entry [91], pH-dependent viral fusion [92], interaction with the capsid protein [93] and viral particle assembly [93]. Neutralizing Abs against conserved regions of structural proteins, including E and prM/M, are widely used due to their specificity. However, Ab use is often challenging due to the enhancement of viral infection in Fcγ receptor-bearing immune cells with subneutralizing doses, as described by the ADE theory [9,94,95]. In addition to Ab therapies, some off-patent drugs and antibiotics have also been tested for repurposing by high-throughput screening, which is beneficial for development time and cost. For instance, prochlorperazine, a dopamine D2 receptor (D2R) antagonist that is used to treat nausea, schizophrenia, migraines, and anxiety, was shown to inhibit viral entry [96].

Considering another perspective, aberrant host immunity also plays vital roles in dengue pathogenesis. Excessive secretion of proinflammatory cytokines (cytokine storm) induced by DENV infection may trigger robust innate and adaptive immune responses, leading to plasma leakage, hemorrhage, and coagulopathy in DHF/DSS patients. Appropriate regulation of host immunity provides different insights into therapeutic targets, including host restriction factors, host dependency factors, and host-mediated pathogenesis pathways [97,98]. Compared with viral targets, host-targeting antiviral approaches are believed to avoid rapid drug resistance or mutations that arise during viral evolution. Since MIF is involved in dengue pathogenesis, the therapeutic potential of blocking MIF to protect against dengue disease has also been studied.

### 5.1. MIF Inhibitors and Neutralizing Antibodies Reduce the Production of Inflammatory Cytokines during DENV Infection

In 2011, Assuncao-Miranda et al. first used the MIF inhibitor ISO-1 and a MIF neutralizing antibody to test the involvement of MIF in DENV3-induced macrophage activation in vitro. Although blocking MIF did not affect viral replication in DENV3-infected human macrophages, both the secretion and mRNA synthesis of TNF-α and IL-6 were reduced. Moreover, prostaglandin E2 (PGE2), a well-known inflammatory mediator in many inflammatory diseases [99], was attenuated as well. Furthermore, they demonstrated that the concentration of IFN-γ in sera, the level of IL-6 in the spleen, and leukocyte infiltration in the lungs of *Mif^−/−^* mice were significantly lower than those in DENV-infected wild-type mice. Most importantly, the study showed delayed mortality in DENV2-infected *Mif*^−/−^ mice. These results suggest that MIF controls amplification of DENV-induced inflammatory responses. Treatment with MIF neutralizing antibodies or inhibitors may provide protection against dengue disease.

### 5.2. Minocycline Attenuates DENV Replication by Targeting MIF

Previously, minocycline, a US Food and Drug Administration (FDA)-approved antibiotic, was found to reduce dengue viral output through downregulation of ERK1/2 activation and upregulation of interferon stimulated genes (ISGs) in Hep G2 cells [100]. In our recent study, we found that minocycline can block not only DENV2-triggered autophagy but also MIF secretion. Autophagy could be activated by MIF through ERK1/2 phosphorylation [59], and the anti-DENV effect of minocycline was abolished in either MIF or LC3-deficient HuH-7 cells during DENV infection. It is possible that the protective effect of minocycline may be due to its ability to block MIF secretion, which suppresses the ERK1/2-autophagy signaling pathway.

In addition, the results showed that minocycline can reduce both MIF RNA transcription and secretion during DENV2 infection, but the mechanism is unclear. Given that MIF secretion can be triggered by the ABC transporter, which is a nonconventional secretory pathway [29], and minocycline is able to inhibit the function of the ABC transporter to block drug–drug interactions at the blood-brain barrier [101], minocycline may disrupt the efflux of MIF via suppression of the ABC transporter upon DENV infection. Moreover, minocycline is reported to reduce the production of TNF-α, IL-6, IL-12, IFN-γ and CCL2 via suppression of the transcription factor NF-κB in the brain, which confers complete protection against JEV in mice [102]. NF-κB binds to the MIF promoter and drives MIF transcription [103], and inhibition of NF-B also blocks DENV infection-induced MIF production in A549 cells [104]; therefore, attenuation of de novo RNA synthesis and secretion of MIF from DENV-infected cells by minocycline treatment may be due to its inhibition of the NF-κB signal pathway and suppression of the ABC transporter, respectively [105]. However, further study is required to clarify these hypotheses.

To further understand whether minocycline can protect against DENV infection in vivo, we found that minocycline treatment reduced the levels of MIF and viremia in sera, as well as attenuated autophagy in murine liver tissue, in AG129 mice. However, the protection of minocycline in AG129 mice was insufficient. To rule out defects in ISG-related protection in this model, which lacks type I and type II IFN receptors, immunocompetent ICR suckling mice were further used. Minocycline only alleviated DENV2-induced neurological symptoms and prolonged the survival rate but did not fully protect against DENV2-induced lethality in suckling mice. It is unclear whether the failure of minocycline to fully protect against DENV2-induced lethality in suckling mice is due to the mouse-adapted strain NGC-N being too virulent for the suckling mice or the intracerebral challenge of NGC-N inducing irreversible damage in the brains of the suckling mice. However, these results were similar to the outcome in DENV2-infected *Mif*^−/−^ mice [48], which suggests that other pathogenic factors induced by DENV infection may also be important for DENV-induced pathogenesis.

### 5.3. Other Therapeutic Approaches to Block MIF and Protect against DENV Infection

MIF plays crucial roles in dengue pathogenesis; however, targeting only MIF secretion and expression seems to be insufficient to provide full protection against DENV infection. As mentioned above, transcription factors, such as HIF-1 and CREB, may also be involved in the increase in MIF expression during DENV infection. It is possible that in addition to MIF, these transcription factors may also induce other pathogenic responses that contribute to disease development during DENV infection [82,83]. On the other hand, although MIF can induce autophagy and facilitate DENV replication in HuH-7 cells, autophagy might play different or even opposite roles in DENV replication in different cells [106]. It has been reported that autophagy plays pro-viral roles in DENV replication in epithelial cells but antiviral roles in immune cells [107]. Therefore, the effect of MIF on the modulation of autophagy and DENV replication should be further systemically investigated in different cells, and the effect of minocycline treatment on DENV infection in different cells, such as immune cells, should be compared. Taken together, these findings suggest that targeting upstream transcription factors that control MIF expression or multiple medication combinations targeting different MIF signaling pathways may help to develop better therapeutic strategies against DHF/DSS in the future.

## 6. Conclusions

DENV-triggered MIF secretion can not only facilitate DENV replication through the regulation of autophagy but also worsen the severity of vascular leak by enhancing endothelial permeability. In addition, MIF may modulate the interaction of different immune cells, which contributes to dengue pathogenesis. Inhibition of MIF secretion and transcription by small molecule drugs such as minocycline could attenuate both autophagy and viral replication. However, targeting MIF by multiple approaches instead of a single approach may provide a better therapeutic alternative against DENV infection for translation from the laboratory to the clinic in the future.
